# Robot-assisted Toupet fundoplication and associated cholecystectomy in symptomatic giant hiatal hernia with situs viscerum inversus—A case report and literature review

**DOI:** 10.1016/j.ijscr.2019.06.038

**Published:** 2019-06-22

**Authors:** Graziano Ceccarelli, Angela Romano, Giuseppe Esposito, Michele De Rosa, Walter Bugiantella, Egidio Miranda, Andrea Fontani, Vito D’Andrea

**Affiliations:** a Department of General Surgery, “San Giovanni Battista” Hospital, Foligno, Italy; b Department of Surgery, Division of General Surgery, “San Donato” Hospital, Arezzo, Italy; c Department of General Surgery, “S. Veneziale”, Isernia, Italy; d Department of Surgical Sciences, Sapienza University of Rome, Rome, Italy

**Keywords:** Robotic surgery, Situs Viscerum Inversus, Toupet fundoplication, Giant hiatal hernia, Mesh placement, Gastro oesophageal reflux disease, Cholecystectomy-case report

## Abstract

**Introduction:**

Giant hiatus hernia is defined as migration of >30% of the stomach with or without other intra-abdominal organs into the chest. Situs Viscerum Inversus is a rare congenital condition in which the major visceral organs are reversed from their normal arrangement; they are translated (completely or partially) on the opposite side of the body. Diagnosis is often incidental. We report a Robot-assisted Toupet fundoplication for a giant hiatal hernia with gastro oesophageal reflux disease and cholelithiasis, in a 63-years-old woman with situs viscerum inversus.

**Presentation of case:**

A 63-year-old woman with Situs Viscerum Inversus was diagnosed with giant sliding hiatus hernia. We performed a Robot-assisted procedure of reduction of hiatal hernia in abdomen and Toupet fundoplication with Bio A mesh placement and gastropexy procedure associated to cholecystectomy. The operation time was of 190 min. The patient was discharged on third postoperative day after X-ray check and he tolerated a solid food.

**Discussion:**

Minimally invasive surgery represents, nowadays, the standard approach for hiatal hernia and cholelithiasis.

**Conclusion:**

In challenging cases as the giant hernias ad rare anomaly as situs viscerum inversus, the surgical treatment can be facilitated by the use of robotic technology.

## Highlights

• All symptomatic paraesophageal hiatal hernias should be repaired, particularly those with acute obstructive symptoms or which have undergone volvulus.
• Laparoscopic hiatal hernia repair is as effective as open transabdominal repair, with a reduced rate of perioperative morbidity and with shorter hospital stays. It is the preferred approach for the majority of hiatal hernias.
• Robotic Assisted Giant-Paraesophageal Hernia repair remain technically challenging predominantly in the dissecation of the hernia sac from the posterior mediastinum.
• The robotic platform have the same benefits of the laparoscopic approach in terms of complication rate, total surgical time, and hospital length of stay and in particular case is superior.

## 1 Introduction

Situs Viscerum Inversus (SVI) with dextrocardia was described first in 1643 by Marco Aurelio Severino and later in human by Matthew Baillie [[1], [2]], it is an uncommon condition occurring in about 0.01% of the population and with equal frequency in males and females [4,5]. It is characterized by transposition of abdominal viscera through the sagittal plane, that may be complete or partial: the complete one (totalis) affects thoracic organs, as well as the abdominal organs, and dextrocardia. The anomaly may be associated to congenital cardiac defects in 10% of cases and Kartagener’s syndrome or primary ciliary dyskinesia. The patients may have normal longevity as compared to the normal population [6,7]. The diagnosis of the condition is often incidental during abdominal study for different diseases, ultrasonography, but above all CT-scan (wit 3-D reconstruction) may be essential for accurate anatomical documentation preliminary to the surgical technique.

Surgery in patients with SVI remains extremely rare and represents a technical challenge for the surgeon. In 1991, Campos and Sipes [8], were the first to report a successful laparoscopic cholecystectomy in a patient with Situs Inversus Totalis. A few cases of laparoscopy on patients with SIT demonstrated that minimally invasive approach is not a contraindication, although may be a challenging situation.

Nissen-360° and Toupet-270° fundoplication represent two different surgical approaches for treating hiatal hernia and gastro-oesophageal reflux disease (GERD). Studies comparing these two procedures demonstrated they are equally effective in restoring the lower oesophageal sphincter function and provide similar long-term control of GERD with no difference in dysphagia [9] although laparoscopic Nissen fundoplication results in decreased EGJ distensibility in patients with GERD compared to partial Toupet fundoplication [10].

Laparoscopic fundoplication for hiatal hernia in patients with SVI is rarely reported [11,12]. Although the surgical procedure is the same for the other patients, right-handed surgeons continue to use the dominant hand for dissection and principal movements while non-dominant hand for the traction. Robotic surgery may overcome the limits of laparoscopy, makes the use of the left and right hand similar, allows the surgeon performing the same procedures using left or right hand with the same safety and precision.

In this case report, we describe a robotic Toupet fundoplication for giant hiatal hernia with associated cholecystectomy in a patient with SVI. To the best of our knowledge, this is the first reported case of Robotic-assisted Toupet fundoplication with associated cholecystectomy in a patient affected by giant hiatal hernia, cholelithiasis and Situs Viscerum Inversus.

The work has been reported in line with the SCARE criteria [13].

## 2 Presentation of case

A 63-year-old woman with partial Situs Viscerum Inversus (without dextrocardia) was diagnosed with giant sliding hiatus hernia and cholelithiasis. The symptoms were typical: heartburn, chest pain, burping and atypical respiratory symptoms as coughing and asthma. The preoperative evaluations was: gastrointestinal endoscopy (EGD), a barium swallow radiography, an intraluminal 24-H pH measurement and oesophageal manometry, abdominal ultrasound, chest and abdominal CT scan with contrast. The diagnosis of Situs Viscerum Inversus was on CT scan exam, and previously unknown. Anatomical variation has to be identified in the preoperative study to avoid intraoperative risks of iatrogenic injures during dissection and exposure of the anatomical structures.

**Fig. 1:**
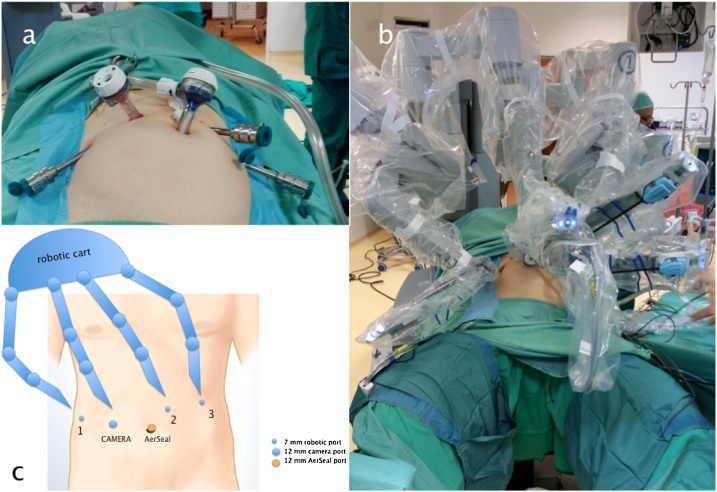
Patient Positioning and Port Placement for Robot-Assisted Surgery.

A robot-assisted reduction of hiatal hernia in abdomen and Toupet fundoplication was performed, a Bio A mesh was placed as hiatoplasty reinforcement and gastropexy (to reduce the post-operative recurrence risk); the cholecystectomy was performed as well as the patient was affected by cholelithiasis. The patient was discharged on third postoperative day after X-ray check and he tolerated a solid food. The operation technique in SVI is quite specular to the conventional technique for robot-assisted fundoplication. The patient was placed in French position, with the surgeon standing between the patient’s legs and robotic cart from the head/right shoulder. We used a 4-arms cart with the 4th arm from the left side (Fig. 1).

Carbon dioxide pneumoperitoneum was established using Veress’ needle technique; a 12-mm incision in the right abdominal quadrant area over-umbilical line was made to insert the trocar for the camera; the ports were placed in a configuration that was roughly the mirror image of our usual fundoplication procedure [13]. The first 12 mm port for the camera was inserted into the abdominal cavity in right ipocondrium, about 2 cm above umbilical line and four more trocars were inserted. After the internal organs of abdominal cavity examination we proceeded with the insertion of three more trocars under direct view. Trocars were placed in epigastric region, left hypocondrium and left lumbar region. The other robotic trocar and auxiliary AirSeal trocar placed.

**Fig. 2:**
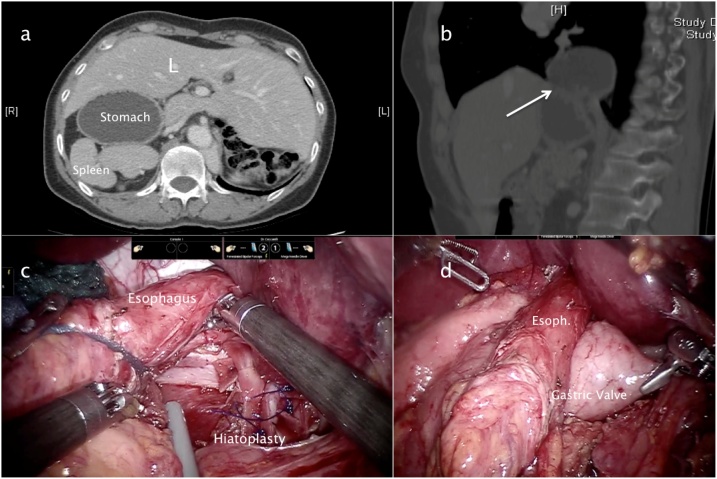
a–b) CT scan images, c) Posterior Hiatoplasty, d) Toupet fundoplication.

Cholecystectomy was first performed. The right hepatic lobe lifted to expose the hiatus. The procedure was carried out in the standard fashion with trans-hiatal dissection of the oesophagus for about 6 cm distally. Reduction of the stomach into the abdomen and the right and left crura exposed, a para-hernia lipoma removed and oesophagus encircled using the articulated tips of robotic instruments and distal oesophagus prepared for about 6–8 cm into the mediastinum, removing the hernia sac. The right side of stomach dissected, no typical short gastric vessels were found, as the spleen was represented by two fetal presentation with a vascular supply from the kidney vessels (Fig. 2).

The left pillar dissected from the cava, the hiatoplasty performed using three stitches with pledgets and a Bio A mesh placed and sutured above the crura plane. Closure of the crura and a posterior partial fundic wrap (Toupet technique) was done with 3/0 Prolene (Prolene, Ethicon Inc., Johnson and Johnson) stitches tied intra-corporeally with hand made pledgets, using pieces of BioA prothesis.

Finally two trans parietal 0-Vycril stitches for the gastropexy between antrum and abdominal wall. The gallbladder was removed with an Endo-bag through the 10-mm operating port; and a drain placed near the hiatal area. The total operative time was 190 min. The patient was discharged on third postoperative day after a contrast upper GI X-ray exam and an oral semi-solid diet.

The operation time (cholecystectomy and fundoplication) was 190 min with no intraoperative complications. The postoperative course was uneventful, and the patient was discharged on postoperative day 3.

## 3 Discussion

Giant hiatal hernia is defined as migration of >30% of the stomach with or without other intra-abdominal organs into the chest. Any patient with a giant hiatal hernias (GHH) should be considered a surgical candidate. The potential risk of incarceration and strangulation is quoted as an indication for surgery. The principles of GHH repair include reducing the hernia, complete excision of hernia’s sac, crural repair and an antireflux procedure [14]. The recurrence rate after laparoscopic repair of GHH is about 30% [15] and to prevent recurrence many authors proposed a mesh to strengthen the crural repair, gastropexy, and Collis gastroplasty [16,17]. Nowaday the gold standard technique treatment of hiatal hernia and gastro-oesophageal reflux disease is a minimally invasive approach.

Situs viscerum inversus (SVI) is a rare autosomal recessive congenital condition of the left/right organ symmetry, it develops in the early stages of the embryonic life, although it could be X-linked. It is due to the mutation of the DNAH11 region, encoding a gene called dynein. A micro-tubule-based motor, involved in the determination of left/right-handed asymmetry. It occurs at an incidence of one in every 4,000–8,000 people. It is characterized by the transposition of the abdominal and/or thoracic organs, but it does not affect health or life expectancy. It is a condition often detected accidentally during a radiological examination [18].

Only a few cases are reported in literature about treatment of hiatal hernia in situs viscerum inversus using a laparoscopic approach [11,12,[19], [20], [21], [22], [23]], but no cases to our knowledge treated by robotic-assisted approach. As the condition and treatment refer to a functional non-oncologic disease, it is very important to select the surgical indications for these patients. If we want to perform minimally invasive approach, an exact mirror image of the usual technique may not be so easy, especially for right-handed surgeons, surgeon experience is required about laparoscopic skills but the procedure may represent a very challenging situation [24].

A literature review about robot-assisted surgery in patients affected by SVI includes distal gastrectomy with D1 lymph node dissection for gastric cancer or anterior resection for rectal cancer, demonstrating this technology may be safe in oncologic surgery too [25].

The advantages of robotic technology are: high definition and 3D view, very precise instruments’ movement and the articulated tips; the left hand may be used as the right with the same precision; it allows performing dissection, sutures, and movements compared to conventional laparoscopic view and instruments. A few series of robot-assisted fundoplication for hiatal hernia and GERD has been reported demonstrating some advantages over conventional laparoscopy only for giant hiatal hernia and redo-surgery, but with longer operative time and higher costs [13,26,27].

About the technical aspects, the aim of anti-reflux surgery is to create an anti-reflux mechanism at the oesophago gastric junction. The two most common types of fundoplications are the 360 ° Nissen and the 270° Toupet: some studies demonstrated a significantly major distensibility at gastro-oesophageal junction after partial fundoplication [28]. The application of mesh-reinforced hiatal closure has been proposed to reduce recurrence rates in comparison with primary suture repair. The use of Gore Bio A^®^ mesh, a synthetic absorbable mesh, it was not the first time, hasn’t the risk of conventional prosthesis complications, especially oesophageal erosion [29]. The mesh was secured with 3/0 vicryl stitches, the mesh is a biocompatible synthetic polymers, gradually absorbed by the body. The tissue scaffold is replaced with type I collagen and subsequently dissolves over 6 months [30]. Gastropexy technique is in our experience quite always performed in giant hiatal hernia with the aim to reduce the recurrence rate described in literature about 30%. The mechanism of recurrence is poorly understood [16]. Finally use of AirSeal device to keep the surgical field stable using low intra-abdominal pressure (about 8 mm HG) reduces the risks of intra and postoperative complications (PNX and postoperative pain). Anyway experience in gastro oesophageal junction surgery and skills in robotic device use are required to the surgeon and surgical team, although in robotic surgery the learning curve period is considered shorter compared to laparoscopic one.

## 4 Conclusion

Symptomatic giant hiatal hernia treatment with Toupet fundoplication and cholecystectomy are nowaday performed safely by laparoscopic approach. Our case report confirms that robot-assisted surgery, although high costs, may be very useful and safe in selected cases as challenging situations, as in the case of giant hiatal hernia in situs viscerum inversus that we present here. Robotic surgery allows performing a more fine and precise anatomical dissection and difficult sutures, with a better anatomical view. Anyway experience in both oesophageal and robotic surgery is required for similar situations.

## Conflicts of interest

None.

## Sources of funding

None.

## Ethical approval

This kind of study is automatically exempt from requiring ethics approval in our Institution.

## Consent

Written informed consent was obtained from the patient for publication of this case report and accompanying images. A copy of the written consent is available for review by the Editor-in-Chief of this journal on request.

## Author contribution

Angela Romano: participated substantially in conception, design, and execution of the study and in the analysis and interpretation of data; also participated substantially in the drafting and editing of the manuscript.

Graziano Ceccarelli: participated substantially in conception, design, and execution of the study and in the analysis and interpretation of data; also participated substantially in the drafting and editing of the manuscript.

Giuseppe Esposito: participated substantially in conception, design, and execution of the study and in the analysis and interpretation of data; also participated substantially in the drafting and editing of the manuscript.

Michele De Rosa: participated substantially in conception, design, and execution of the study and in the analysis and interpretation of data.

Walter Bugiantella: participated substantially in conception, design, and execution of the study and in the analysis and interpretation of data.

Egidio Miranda: participated substantially in conception, design, and execution of the study and in the analysis and interpretation of data.

Andrea Fontani: participated substantially in conception, design, and execution of the study and in the analysis and interpretation of data.

Vito D’Andrea: participated substantially in conception, design, and execution of the study and in the analysis and interpretation of data.

## Registration of research studies

N/A.

## Guarantor

Graziano Ceccarelli, MD.

## Provenance and peer review

Not commissioned, externally peer-reviewed.
